# Pregnancy Outcomes among Women with Graves’ Hyperthyroidism: A Retrospective Cohort Study

**DOI:** 10.3390/jcm10194495

**Published:** 2021-09-29

**Authors:** Panwad Harn-a-morn, Prapai Dejkhamron, Theera Tongsong, Suchaya Luewan

**Affiliations:** 1 Department of Obstetrics and Gynecology, Faculty of Medicine, Chiang Mai University, Chiang Mai 50200, Thailand; harnamorn.pan@gmail.com (P.H.-a.-m.); theera.t@cmu.ac.th (T.T.); 2 Department of Pediatrics, Faculty of Medicine, Chiang Mai University, Chiang Mai 50200, Thailand; prapai.dej@cmu.ac.th

**Keywords:** abortion, low birth weight, preeclampsia, preterm birth, thyrotoxicosis

## Abstract

Objective: The primary objectives of this study are to compare the rates of preterm birth; fetal growth restriction and low birth weight between the following groups: (1) pregnant women treated for thyrotoxicosis and low-risk pregnancies; (2) between pregnant women with thyrotoxicosis with no need of medication and low-risk pregnancies; and (3) between those treated with MMI and PTU. Methods: The medical records of singleton pregnancies with thyrotoxicosis were comprehensively reviewed. Low-risk pregnancies matched for age and parity were randomly recruited as controls. The obstetric outcomes were compared between both groups; the outcomes of various subgroups of the thyrotoxicosis group were also compared. Results: A total of 408 pregnant women with thyrotoxicosis were recruited. Compared with the controls; the women of the thyrotoxicosis group had significantly higher rates of low birth weight (LBW) (23.7% vs. 17.7%; *p*: 0.036), preterm birth (19.3% vs. 12.3%; *p*: 0.007), preeclampsia (8.5% vs. 4.4%; *p*: 0.019) and cesarean section (21.5% vs. 16.0%; *p*: 0.046). In the thyrotoxicosis group; 67; 127; and 158 patients were treated with MMI; PTU and no anti-thyroid drug (ATD), respectively. All obstetric outcomes were comparable between the women treated with PTU and those with MMI; and between the controlled and uncontrolled groups. However, women who needed ATD had significantly higher rates of LBW and preterm birth than those without medications. Conclusions: Thyrotoxicosis, whether treated or not needing ATDs, was significantly associated with an increased risk of adverse pregnancy outcomes. Also, active disease, indicated by the need for ATD significantly increased the risk of such adverse outcomes; whereas the patients treated with MMI or PTU had comparable adverse outcomes.

## 1. Introduction

Thyrotoxicosis is a condition involving the thyroid gland producing and secreting excessive amounts of thyroid hormone. Thyrotoxicosis occurs in 0.2% of pregnancies [1]. The most common cause is Graves’ disease, accounting for 90% of cases.

Pregnancy complicated with poorly controlled thyrotoxicosis is associated with an increased risk of adverse pregnancy outcomes, including, spontaneous abortion, preterm birth, low birth weight, stillbirth, fetal hypothyroid and hyperthyroid [2,3]. Moreover, maternal complications such as preeclampsia, heart failure and thyroid storm are also increased [4,5]. Therefore, treatment is important to control the disease to reduce such adverse pregnancy outcomes. However, in actual practice, most cases of pregnancies with thyrotoxicosis are under control, and adverse pregnancy outcomes among patients treated for thyrotoxicosis have not been well-established [2,3]. For example, one study showed that treated hyperthyroidism was not associated with adverse perinatal outcome but it was an independent risk factor for cesarean delivery [6]. Whether or not women treated with anti-thyroid drugs (ATD) are at a higher risk of adverse outcomes is yet to be elucidated. Moreover, regarding the two most commonly used ATDs, methimazole (MMI) and propylthiouracil (PTU), there is controversy about which one is better for pregnant women. The number of studies comparing adverse pregnancy outcomes between the two drugs is limited and they are all retrospective [7,8]. Both ATDs have some disadvantages. MMI is potentially teratogenic and PTU can be hepatotoxic. MMI has been documented to increase the risk of dysmorphic facies and aplasia cutis [9,10,11]. Also, PTU, though considered a safer ATD in terms of teratogenicity, was demonstrated to be associated with congenital anomaly in 2–3% of cases [10]. Additionally, in 2010, FDA called attention to the risk of hepatotoxicity in patients exposed to PTU [12,13]. However, though abnormalities of liver blood tests are common in newly diagnosed and untreated cases, the recent meta-analysis supports that there is a high chance of safely normalizing elevated transaminases up to fivefold above the upper normal limits with the use of ATDs in the treatment of hyperthyroidism, though this study was conducted on non-pregnant patients [14]. It, it is recommended that ATD during pregnancy should be administered at the lowest effective dose of MMI or PTU, targeting maternal serum-free T4 at the upper limit or slightly above the reference range [2]. Thus, several cases received no ATDs throughout pregnancy or during most part of pregnancy courses, especially in cases of mild clinical symptoms. To our best knowledge, this group of patients has not been thoroughly evaluated for risk of adverse pregnancy outcomes.

The primary objectives of this study are to compare the rates of preterm birth, fetal growth restriction and low birth weight between the following groups: (1) pregnant women treated for thyrotoxicosis and low-risk pregnancies, (2) between pregnant women with thyrotoxicosis with no need of medication and low-risk pregnancies, and (3) between those treated with MMI and PTU.

## 2. Materials and Methods

The present study is a retrospective cohort study based on a prospective database; which was conducted at Maharaj Nakorn Chiang Mai Hospital, Thailand, a tertiary care center and teaching school, and covers a period of 1994 to 2018. This study was ethically approved by the Institutional Review Board of the Faculty of Medicine, Chiang Mai University. The database of Maternal–Fetal Medicine unit was accessed to retrieve the records of singleton pregnancies with thyrotoxicosis, and the medical records were comprehensively reviewed. On the development of the database, all consecutive cases of pregnancies diagnosed with thyrotoxicosis were reviewed and prospectively recorded at the time of discharge from the hospital after delivery. The inclusion criteria of this thyrotoxicosis group are as follows: (1) singleton pregnancy, (2) diagnosis of thyrotoxicosis, either before or during pregnancy, and being taken care of by our endocrinologists (Endocrinology Unit, Department of Internal Medicine, Chiang Mai University), which was defined as a decreased TSH level an increased free T4. The trimester-specific reference ranges for TSH, recommended by the American Thyroid Association were used: first trimester, 0.1–2.5 mIU/L; second trimester, 0.2–3.0 mIU/L; third trimester, 0.3–3.0 mIU/L. (3) attending prenatal care and giving birth at our hospital, (4) no serious medical diseases such as pre-gestational diabetes, heart diseases, etc., and (5) known final obstetric outcomes. Of note, the study group included only thyrotoxicosis caused by Graves’ disease, whereas gestational thyrotoxicosis, Hashitoxicosis, toxic goiters, drug-induced and LT4 excess were not included. The obstetric database of the department, covering the same period as the thyrotoxicosis group, was accessed to randomly retrieve the records of low-risk pregnancies, as the control group with a control-to-case ratio of 1:1, matched for maternal age within a range of two years, parity (with a difference of not more than one), and gestational age at the first visits within two weeks. The controls were validated using the same inclusion criteria as the thyrotoxicosis group but had no thyrotoxicosis and were taken care as low risk pregnancies at Maharaj Nakorn Chiang Mai Hospital. The pregnancies with complicated with other medical diseases and incomplete medical records were excluded.

The full medical records of the pregnant women with thyrotoxicosis were comprehensively reviewed; demographic, clinical and laboratory data were digitally recorded. Laboratory parameters included complete blood counts, thyroid function tests, and thyroid antibodies. 

Patients in the thyrotoxicosis group were sub-categorized into three groups: MMI, PTU, and no medication, in cases of using the two ATDs were categorized into the group of drugs that was used most of the time during pregnancy. Note that some patients had no medication since free T4 levels were slightly above normal range or the symptom is mild (i.e., pulse rate of 100–105 bpm), as recommended by the American College of Obstetricians and Gynecologists [2]. The judgement on medications used was based on our endocrinologists. Additionally, patients in the thyrotoxicosis group were also categorized into two subgroups, controlled and uncontrolled, based on thyroid function tests validated by the endocrinologists. Controlled thyrotoxicosis was defined as no clinical symptoms and free T4 levels of slightly above or in the high-normal range, in most parts of the pregnancy courses. Uncontrolled thyrotoxicosis was defined when the patients had significant symptoms or often abnormal free T4 levels in spite of taking ATDs. Controlled status was arbitrarily judged by the author team. The control status was validated in three periods of pregnancy; early second trimester, early third trimester and before delivery.

The main adverse outcomes were the rates of preterm birth (delivery before 37 complete weeks of gestation), fetal growth restriction (birth weight of less than 10th percentile of reference ranges), and low birth weight (birth weight of less than 2500 g). The additional outcomes included the rates of abortion (ending up at 24 weeks of gestation or earlier) and stillbirth, preeclampsia (defined as a new onset of hypertension and either proteinuria; 24-h urine protein > 300 mg, or end-organ dysfunction after 20 weeks of gestation in a previously normotensive woman), cesarean section (in actual practice; performed as obstetric indications, not based on the status of thyrotoxicosis), low Apgar scores (less than a score of 7 at 5 min), antepartum hemorrhage and postpartum hemorrhage. The primary outcomes were comparisons of the above-mentioned outcomes between (1) the thyrotoxicosis group and control groups, (2) the control (no thyrotoxicosis) group and the group of patients with thyrotoxicosis with no need of ATDs. (3) Among the thyrotoxicosis group, the subgroups of patients with need and no need of ATDs, and (4) Among the thyrotoxicosis group, the subgroups of patients with treatment with PTU and those with MMI. 

### Statistical Analysis

The statistical analysis was performed using the statistical software SPSS version 21.0 (IBM Corp. Released 2012; IBM SPSS Statistics for Windows, Version 21.0. Armonk, NY, USA). For comparison of the various pregnancy outcomes between the study and control groups, the Chi-square test, as well as relative risk with 95% CI, T-Test and Mann-Whitney U test, were used where appropriate. *p*-values of less than 0.05 were considered statistically significance.

## 3. Results

A total of 408 pregnant women with thyrotoxicosis, meeting the inclusion criteria were recruited as the thyrotoxicosis group, and 408 matched controls were also recruited. Patients in the thyrotoxicosis group were categorized into three groups based on the main type of ATDs used: PTU (*n* = 128), MMI (*n* = 67), no ATD treatment (*n* = 158) and heterogeneous data (*n* = 55; mixed use of ATDs, or switched to each other). Most baseline characteristics of the thyrotoxicosis group and the control group were not significantly different, as presented in Table 1.

Compared with the controls, the women of the thyrotoxicosis group had significantly higher rates of low birth weight (LBW) (23.7% vs. 17.7%; *p*: 0.036), preterm birth (19.3% vs. 12.3%; *p*: 0.007), preeclampsia (8.5% vs. 4.4%; *p*: 0.019) and cesarean section rate (21.5% vs. 16.0%; *p*: 0.046), as presented in Table 2. Likewise, mean birth weight and gestational age in the thyrotoxicosis group were also significantly lower than those in the control group. The rate of fetal growth restriction (FGR) tended to be higher in the thyrotoxicosis group but did not reach statistical significance (11.5% vs. 7.9%; *p*: 0.085). Other adverse pregnancy outcomes (low Apgar scores, stillbirth, obstetric hemorrhage and fetal anomaly) were not significantly different. Interestingly, patients with thyrotoxicosis with no need for ATD who still had a significantly higher risk of adverse pregnancy outcomes when compared to the controls, as presented in Table 2.

In the study (thyrotoxicosis) group, when sub-grouped based on status of controlling the disease, the rates of all adverse pregnancy outcomes in the subgroup of controlled and uncontrolled disease were not significantly different in all three periods of evaluation as presented in Table 3.

Considering the thyrotoxicosis group, when sub-grouped based on ATD treatment, the patients requiring ATD had a significantly higher risk of preterm birth (15.3% vs. 20.8%, *p*: 004), and low birth weight (19.2% vs. 29.2%, *p*: 0.033), whereas the rates of other adverse outcomes were comparable as presented in Table 4. Likewise, mean birth weight and gestational age in the patients requiring ATD were also significantly lower than those requiring no ATD. In further subgroup analysis, all obstetric outcomes were comparable between the women treated with PTU and those with MMI, as presented in Table 4.

## 4. Discussion

This study demonstrated that pregnant women with thyrotoxicosis had an increased risk of preterm birth, low birth weight, preeclampsia and cesarean section compared with low-risk pregnancies. The results are consistent with those of most previous studies [1,2,4,5]. Nevertheless, new insights gained from this study are as follows: (1) Thyrotoxicosis, though treated and under control, was significantly associated with an increased risk of adverse pregnancy outcomes. (2) Active disease, indicated by the need for ATD, significantly increased the risk of such adverse outcomes. (3) Regarding pregnancies with thyrotoxicosis, women who received MMI and PTU treatments had comparable adverse outcomes, and no serious complications associated with medications were observed in both groups. (4) The cases that needed no ATD and were well-controlled, still had higher rates of adverse pregnancy outcomes, compared with low-risk pregnancies, indicating that treatment is needed even in cases with mild symptoms or free T4 levels just only slightly above the reference range.

Interestingly, the cases that needed medications, either PTU or MMI, had a higher rate of adverse pregnancy outcomes compared with low-risk pregnancies. The findings imply that more severe cases had a higher risk of adverse outcomes even though they had been treated with ATD. The effects of the disease on adverse outcomes could not be completely prevented by ATD. However, it is possible that both medications might have had some effects on adverse pregnancy outcomes. However, no study has explored whether the severity of the disease or medications mainly resulted in the adverse outcomes.

American Thyroid Association (ATA) strongly recommends PTU for treatment through 16 weeks of pregnancy [3]. However, due to low-quality evidence, ATA strongly recommends that pregnant women receiving MMI who are in need of continuing therapy during pregnancy should be switched to PTU as early as possible. If ATD therapy is required after 16 weeks of gestation, it remains unclear whether PTU should be continued or the therapy changed to MMI. Both medications are associated with potential adverse effects and switching, which potentially may lead to a period of less-tight control and possibly increasing adverse effects [9]. Thus, because of insufficient evidence, ATA currently has no recommendation regarding switching anti-thyroid drugs. Accordingly, more information on the safety of the two ATDs is needed to accumulate in the literature. 

Surprisingly, the overall rate of adverse pregnancy outcomes (preterm birth, fetal growth restriction, low birth weight, preeclampsia and low Apgar scores) of the controlled and uncontrolled disease groups was comparable, different from those of previous reports [4]. The reasons for the contradictory results are unclear. It is possible that the sample size of the uncontrolled group was relatively small, lacking enough power to express the difference, if it exists. On the other hand, all cases treated with ATD had a higher risk of adverse outcomes than cases requiring no medications as mentioned above. Cases of ATD usually had active disease, which was either well- or poorly- controlled. The overall findings suggest that active disease is an important factor associated with adverse pregnancy outcomes.

Notably, the group of no medication during pregnancy still had higher rates of adverse pregnancy outcomes. Most of them had mild hyperthyroidism or minimal clinical disease, and avoidance of all ATD therapy was possible, as the most commonly recommended to avoid potential teratogenic effects. Our findings challenge this recommendation. Whether or not mild thyrotoxicosis during pregnancy should be treated with ATD is yet to be explored to weigh between the adverse obstetric outcomes associated with no treatment and the teratogenic effects of ATDs. Theoretically, in patients with mild clinical disease, ATD should be avoided in the first trimester and continued thereafter. If needed, PTU is generally favored in the first trimester and switching to MMI to decrease the risk of maternal hepatotoxicity thereafter.

The strengths of this study are as follows; (1) We did not just compare the adverse outcomes between the group of thyrotoxicosis and normal controls, but also compared between subgroups, such as controlled and uncontrolled groups as well as patients in need of medications and those not in need of ATDs. (2) We compared the outcomes between the groups treated with MMI and PTU, which has rarely been performed in previous studies. (3) Cases with confounding factors for adverse pregnancy outcomes existing before pregnancy such as medical diseases like chronic hypertension or pregestational DM were excluded.

The weaknesses of this study are as follows: (1) Although the sample might be adequate to compare the outcomes between the thyrotoxicosis group and the control group, it was relatively small for comparison of the outcomes between the two groups of ATDs. The teratogenic effects of ATDs, which are a great concern, could not be evaluated since the sample size is too small for such a rare occurrence. (2) Thyroid receptor antibody was not available in most cases. (3) Some definitions used in this study were relatively arbitrary or subjective and probably less reliable. For example, categorization of the disease into controlled or uncontrolled was somewhat difficult in many cases due to the fact that the case categorized as controlled might have a long history of been uncontrolled during pregnancy before been brought under control in late pregnancy, leading to poor outcomes in spite of good control. Moreover, the ATD group might be inconsistent. The doses of drug used could be varied among the patients according to the severity of the disease or doctors’ attitude. The three groups of ATDs (MMI, PTU, and no ATD) were classified according to the ATD they took most of the time during pregnancy (not all the time). Some cases might take PTU for only a short period in early pregnancy and then switch to MMI throughout the remaining period of pregnancy. 

## 5. Conclusions

The interesting insights gained from this study are that thyrotoxicosis, though treated, was significantly associated with an increased risk of adverse pregnancy outcomes. Our evidence shows that active disease, reflected by the need for ATD significantly increased the risk of such adverse outcomes. Moreover, even under control with ATD, poor pregnancy outcomes were still significantly higher than those with no ATD. No ATD prior to pregnancy and no need for ATD throughout pregnancy seem to lower the risk, minimally, but it is still significantly higher than the controls. Our findings raise an important concern regarding whether the ATDs given should be as low as possible to control the disease with acceptable mild clinical symptoms of thyrotoxicosis or whether the upper normal limit of thyroid function test parameters should be maintained since even in cases of no ATDs, adverse outcomes were still higher than normal controls. Also, it should be emphasized that the concerns with anti-thyroid drugs are focused primarily on the embryopathy effects on the fetus. Finally, with limited data, MMI and PTU may result in the same rates of adverse pregnancy outcomes.

## Figures and Tables

**Table 1:** Baseline characteristics of pregnancies in the thyrotoxicosis group (pregnancies with thyrotoxicosis) and the control group.

Outcome	Thyrotoxicosis Group (*n* = 408)	Control Group (*n* = 408)	*p*-Value
Mean maternal age; Year ± SD	29.0 ± 6.0	29.0 ± 5.7	0.981
Number of antenatal care visits: No. ± SD	9.7 ± 2.9	9.1 ± 3.4	0.522
Parity:			0.834
Nulliparous women	211 (51.7%)	208 (51.0%)	
Parous women	197 (48.3%)	200 (49.0%)	
Education:			0.920
High school or lower	71 (22.3%)	68 (22.7%)	
Higher education (higher than high school; including vocational/high vocational certificate, Bachelor of Arts, Master of Arts/Doctor of Philosophy)	247 (77.7%)	232 (77.3%)	
Residency:			0.034
Chiang Mai	178 (56.0%)	193 (64.3%)	
Others (nearly all in the northern part of Thailand)	140 (44.0%)	107 (35.7%)	

**Table 2:** Comparisons of the pregnancy outcomes between pregnancies with thyrotoxicosis vs. controls and pregnancies with thyrotoxicosis with no ATD vs. controls.

Outcomes	Control *n* = 408	Case: Thyrotoxicosis *n* = 408	Relative Risk (95% CI)	*p* Value	Case: Thyrotoxicosis with No ATD *n* = 158	Relative Risk (95% CI)	*p* Value
Quantitative data	Mean ± SD	Mean ± SD			Mean ± SD		
Gestational weeks at delivery	37.9 ± 2.7	37.1 ± 3.8	-	0.001	37.0 ± 4.0	-	0.004
Birth weight (grams)	2874 ± 601	2778 ± 728	-	0.042	2767 ± 736	-	0.081
Categorical data	*n*/*N* (%)	*n*/*N* (%)			*n/N* (%)		
Abortion	2/408 (0.5%)	8/408 (2.0%)	4.0 (0.8–18.7)	0.056	4/158 (2.5%)	5.1 (0.9–27.6)	0.035
Antepartum hemorrhage	16/406 (3.9%)	14/400 (3.5%)	0.9 (0.4–1.7)	0.741	8/156 (5.1%)	1.3 (0.6–2.9)	0.533
Preeclampsia	18/406 (4.4%)	34/400 (8.5%)	1.9 (1.1–3.3)	0.019	14/156 (9.0%)	2.0 (1.0–4.0)	0.038
Cesarean delivery	65/406 (16.0%)	86/400 (21.5%)	1.3 (1.0–1.8)	0.046	28/156 (17.9%)	1.1 (0.7–1.7)	0.580
Preterm birth < 37 wk	50/406 (12.3%)	77/399 (19.3%)	1.6 (1.1–2.2)	0.007	30/155 (19.4%)	1.6 (1.0–2.4)	0.033
Stillbirth	8/406 (2.0%)	6/400 (1.5%)	0.8 (0.3–2.2)	0.609	3/156 (1.9%)	1.0 (0.3–3.6)	0.971
Postpartum hemorrhage	10/408 (2.5%)	8/408 (2.0%)	0.8 (0.3–2.0)	0.634	2/158 (1.3%)	0.5 (0.1–2.3)	0.371
Fetal growth restriction	32/406 (7.9%)	45/392 (11.5%)	1.5 (0.9–2.2)	0.085	16/154 (10.4%)	1.3 (0.7–2.3)	0.344
Low birth weight	72/406 (17.7%)	94/396 (23.7%)	1.3 (1.0–1.8)	0.036	40/158 (25.3%)	1.4 (1.0–2.0)	0.043
Low Apgar	11/406 (2.7%)	16/396 (4.0%)	1.5 (0.7–3.2)	0.296	9/155 (5.8%)	2.1 (0.9–5.1)	0.077
Fetal anomaly	9/382 (2.4%)	7/408 (1.7%)	0.7 (0.3–1.9)	0.523	2/158 (1.3%)	0.5 (0.1–2.4)	0.405

**Table 3:** Comparisons of the major adverse pregnancy outcomes in the thyrotoxicosis groups between the control levels at early second trimester, early third trimester and before delivery.

	Early Second Trimester			Early Third Trimester			Before Delivery		
Outcomes	Well- Controlled (*n* = 97)	Uncontrolled (*n* = 106)	*p* Value	Well- Controlled (*n* = 136)	Uncontrolled (*n* = 134)	*p* Value	Well- Controlled (*n* = 153)	Uncontrolled (*n* = 139)	*p* Value
Quantitative data	Mean ± SD	Mean ± SD		Mean ± SD	Mean ± SD		Mean ± SD	Mean ± SD	
Gestational weeks at delivery	37.1 ± 3.4	37.4 ± 3.3	0.584	37.5 ± 2.7	37.5 ± 3.0	0.917	37.2 ± 3.1	37.6 ± 2.9	0.298
Birth weight (grams)	2793 ± 713	2845 ± 719	0.605	2794 ± 675	2858 ± 681	0.436	2766 ± 696	2876 ± 687	0.175
Categorical data	*n*/*N* (%)	*n*/*N* (%)		*n*/*N* (%)	*n*/*N* (%)		*n*/*N* (%)	*n*/*N* (%)	
Preeclampsia	9/97 (9.3%)	10/106 (9.4%)	0.970	10/136 (7.4%)	11/134 (8.2%)	0.793	11/153 (7.2%)	9/139 (6.5%)	0.809
Preterm birth < 37 wk	19/96 (19.8%)	22/106 (20.8%)	0.865	22/135 (16.3%)	33/134 (24.6%)	0.090	25/152 (16.4%)	33/139 (23.7%)	0.120
Fetal growth restriction	8/97 (8.2%)	13/106 (12.3%)	0.348	15/135 (11.1%)	16/132 (12.1%)	0.797	18/151 (11.9%)	16/137 (11.7%)	0.949
Low birth weight	24/97 (24.7%)	28/106 (26.4%)	0.785	32/135 (23.7%)	31/133 (23.3%)	0.939	37/152 (24.3%)	32/138 (23.2%)	0.818
Low Apgar	5/96 (5.2%)	2/106 (1.9%)	0.197	8/134 (6.0%)	5/134 (3.7%)	0.394	9/150 (6.0%)	5/139 (3.6%)	0.342

**Table 4:** Comparisons of the major adverse pregnancy outcomes in the study groups between the patients on ATDs vs. no ATDs and between the patients using PTU vs. MMI in the subgroup of those on ATDs.

Outcomes	No ATD *n* = 158	On ATD *n* = 194	*p* Value	PTU *n* = 128	MMI *n* = 67	*p* Value
Quantitative data	Mean ± SD	Mean ± SD		Mean ± SD	Mean ± SD	
Gestational weeks	37.7 ± 3.1	36.8 ± 3.9	0.027	37.0 ± 4.2	37.2 ± 2.5	0.791
Birth weight (grams)	2943 ± 615	2679 ± 792	0.001	2738 ± 767	2728 ± 719	0.933
Categorical data	*n*/*N* (%)	*n*/*N* (%)		*n*/*N* (%)	*n*/*N* (%)	
Preeclampsia	16/158 (10.1%)	15/186 (8.1%)	0.506	11/128 (8.6%)	4/66 (6.1%)	0.531
Preterm birth < 37 wk	24/157 (15.3%)	45/186 (20.8%)	0.040	26/128 (20.3%)	15/66(22.7%)	0.696
Fetal growth restriction	15/155 (9.7%)	25/184 (13.6%)	0.266	16/126 (12.7%)	6/66 (9.1%)	0.456
Low birth weight	30/156 (19.2%)	54/185 (29.2%)	0.033	31/127 (24.4%)	17/66 (25.8%)	0.837
Low Apgar	5/157 (3.2%)	9/184 (4.9%)	0.429	8/126 (6.3%)	2/66 (3.0%)	0.326

## Data Availability

The datasets analyzed during the current study are available from the corresponding author upon reasonable request.
